# Increased Level of Myeloid-Derived Suppressor Cells, Programmed Death Receptor Ligand 1/Programmed Death Receptor 1, and Soluble CD25 in Sokal High Risk Chronic Myeloid Leukemia

**DOI:** 10.1371/journal.pone.0055818

**Published:** 2013-01-31

**Authors:** Lisa Christiansson, Stina Söderlund, Emma Svensson, Satu Mustjoki, Mats Bengtsson, Bengt Simonsson, Ulla Olsson-Strömberg, Angelica S. I. Loskog

**Affiliations:** 1 Department of Immunology, Genetics and Pathology, Science for Life Laboratory, Uppsala University, Uppsala, Sweden; 2 Department of Medical Sciences, Uppsala University and Department of Hematology, University Hospital, Uppsala, Sweden; 3 Hematology Research Unit Helsinki, Department of Medicine, Division of Hematology, University of Helsinki and Helsinki University Central Hospital, Helsinki, Finland; 4 Section of Clinical Immunology and Transfusion Medicine, Uppsala University Hospital, Uppsala, Sweden; International Center for Genetic Engineering and Biotechnology, India

## Abstract

Immunotherapy (eg interferon α) in combination with tyrosine kinase inhibitors is currently in clinical trials for treatment of chronic myeloid leukemia (CML). Cancer patients commonly have problems with so called immune escape mechanisms that may hamper immunotherapy. Hence, to study the function of the immune system in CML is of interest. In the present paper we have identified immune escape mechanisms in CML with focus on those that directly hamper T cells since these cells are important to control tumor progression. CML patient samples were investigated for the presence of myeloid-derived suppressor cells (MDSCs), expression of programmed death receptor ligand 1/programmed death receptor 1 (PD-L1/PD-1), arginase 1 and soluble CD25. MDSC levels were increased in samples from Sokal high risk patients (p<0,05) and the cells were present on both CD34 negative and CD34 positive cell populations. Furthermore, expression of the MDSC-associated molecule arginase 1, known to inhibit T cells, was increased in the patients (p = 0,0079). Myeloid cells upregulated PD-L1 (p<0,05) and the receptor PD-1 was present on T cells. However, PD-L1 blockade did not increase T cell proliferation but upregulated IL-2 secretion. Finally, soluble CD25 was increased in high risk patients (p<0,0001). In conclusion T cells in CML patients may be under the control of different immune escape mechanisms that could hamper the use of immunotherapy in these patients. These escape mechanisms should be monitored in trials to understand their importance and how to overcome the immune suppression.

## Introduction

Chronic myeloid leukemia (CML) is a myeloproliferative disorder characterized by the Philadelphia chromosome (Ph) [1]. Sokal score predicts the prognosis and divides CML patients into a low (LR), intermediate (IR) or high risk (HR) group [2]. Regardless of Sokal score the standard treatment for CML is tyrosine kinase inhibitors (TKIs). TKIs have profoundly changed the course of the disease with an overall survival of 88 percent and with 63 percent of patients still having optimal response after six years of treatment with the TKI imatinib [3]. So far, the only proven cure for CML is allogeneic stem cell transplantation where the graft-versus-leukemia effect is considered to be of central importance implying immunological mechanisms in the disease control [4], [5]. Lately however, a study with patients discontinuing imatinib has shown that 41 percent of the patients stopping treatment in complete molecular response (CMR) remained in CMR at 12 months follow-up implicating that also imatinib may cure a subpopulation of patients [6]. With the aim of increasing cure rates and make it possible for patients to discontinue treatment, TKI therapies are currently evaluated in combination with immune modulators in studies that have shown promising results [7], [8], [9], [10]. Because of the interest of immune modulators in CML a better understanding of the underlying cancer-associated immune escape mechanisms in CML is warranted.

Cancer patients are known to have a suppressed anti-tumor response that complicates the development and use of immunotherapy. Myeloid-derived suppressor cells (MDSCs) are a heterogeneous cell population of myeloid cells that is known to increase in many cancers [11] and has been shown to be more suppressive in cancer patients than in healthy control subjects (HCs) [12], [13], [14]. MDSCs have the ability to inhibit T cell responses by various mechanisms such as secretion of reactive oxygen species [15] as well as up-regulation of arginase 1 (Arg1) [16]. The increased expression of Arg1 leads to L-arginine starvation which inhibits the immune response by T cell cycle arrest [17]. Since the tumor cells in CML are immature and of myeloid origin their role as potential MDSCs are of interest to investigate.

Tumor cells can suppress immunity by direct contact with immune cells or by secreting immune inhibitory molecules [18]. For example, tumor cells can express programmed death receptor ligand 1 (PD-L1, CD274, B7-H1), a member of the B7-family of co-stimulatory molecules, that acts as a co-inhibitory molecule for T cells by binding the programmed death receptor 1 (PD-1) upregulated on activated T cells [19]. The expression of PD-L1 and PD-1 in cancer patients has been suggested to lead to disease progression due to T cell exhaustion [20]. In CML, Mumprecht et al demonstrated higher PD-1 expression on CD8 T cells compared to CD8 T cells from healthy control subjects. Further, in a mouse model of CML they found PD-L1 expression on leukemic cells and that PD-L1 blockade enhanced survival of CML mice in blast crisis [20]. A secreted molecule, the soluble form of the IL-2 receptor α-chain, soluble CD25 (sCD25) may be an immune inhibitor in hematological malignancies [21]. Originally, elevated levels of sCD25 was associated with lymphocyte activation [22]. However, in hematological malignancies sCD25 is thought to be released from tumor cells and it has been correlated to tumor burden in the patients [23]. Moreover, we have previously shown that sCD25 was released from T regulatory cells in samples from patients with B cell lymphoma [21].

In the present study, newly diagnosed HR and LR CML patients were investigated for the presence and nature of immune escape mechanisms including MDSCs, Arg1, PD-L1/PD-1 and sCD25 in an attempt to map the immune status of CML patients.

## Design and Methods

### Patient samples, samples from control subjects, chronic myeloid leukemia cell lines and ethics statement

Cryopreserved leukapheresis samples from newly diagnosed CML patients (n = 18, patients 1–18 in Table 1) were obtained from Uppsala University Hospital Biobank. Fresh blood from newly diagnosed CML patients (n = 19, patients 11 and 19–36 in Table 1) was obtained from Uppsala University Hospital, section of Hematology. Since only a few persons per year are diagnosed with CML in Uppsala, cryopreserved samples were used to get enough patient material to study. This study was approved by Uppsala Regional Research Ethics Committee and all patients gave their written informed consent (DNr: 2009/288, 2005/164). The cryopreserved leukapheresis samples had been routinely saved in Uppsala University Hospital Biobank and before samples were taken from the biobank written informed consent was obtained from the patients, as approved by the regional ethics review board. At the time the patients gave their informed consent they had all past 18 years of age. As controls, buffy coats from gender- and age matched control subjects (n = 30) were obtained from the blood bank at Uppsala University Hospital. For plasma separation, peripheral blood from control subjects (n = 18) was obtained through the blood bank at Uppsala University Hospital. Blood plasma from CML patients and control subjects was obtained by centrifugation of fresh heparinized blood. Peripheral blood mononuclear cells (PBMCs) from healthy control subjects were separated from buffy coats by ficoll separation (GE Healthcare, Uppsala, Sweden) and cryopreserved in RPMI-1640 supplemented with 40% fetal bovine serum (FBS) and 10% dimethyl sulfoxid (DMSO) (Apoteket AB, Uppsala, Sweden). Red blood cells in buffy coats and fresh CML patient blood were lysed by two times five minutes incubation with red blood cell lysis buffer containing 155 mM NH_4_Cl, 10 mM KHCO_3_ and 0,1 mM EDTA at a pH of 7,4. The white blood cells from buffy coats were cryopreserved as indicated above and the white blood cells from CML patients were cryopreserved in FBS supplemented with 10% DMSO. For fluorescent in situ hybridization (FISH) five fresh CML patients were obtained from Helsinki University central hospital. The CML cell lines K562, CML-T1 and BV-173 (K562 obtained from ATCC Manassas, VA, USA, CML-T1 and BV-173 obtained from Deutsche Sammlung von Mikroorganismen und Zellen, Braunschweig, Germany), all originally from CML patients in blast crisis, were cultured in RPMI-1640 media supplemented with 10% FBS and 1% penicillin/streptomycin (PEST). All cell culture reagents were from Invitrogen (Carlsbad, CA, USA).

**Table 1:** Patient characteristics.

Patient ID	Age^1^	Sex	Sokal risk group	Eutos score	Spleen size (cm)^2^	WBC (10^9^/L)	Platelets ×10^9^/L	PB blasts (%)	PB basophils (%)
1	31	F	High	Low	5	172	365	8	14
2	59	M	High	Low	10	276	586	8	4
3	37	M	High	Low	14	180	247	7	2
4	49	F	High	Low	16	555	588	2	4
5	65	M	High	Low	10	210	285	3	3
6	53	F	High	Low	15	322	276	4	1
7	40	M	High	Low	4	205	824	12	12
8	64	F	High	Low	0	144	259	5	6
9	37	M	High	Low	15	219	425	4	5
10	18	M	High	Low	21	268	488	8	1
11	29	M	High	Low	20	340	304	6	10
12	66	M	Low	Low	0	90	205	0	4
13	22	M	Low	Low	3	220	332	2	2
14	39	M	Low	Low	2	141	285	1	3
15	50	M	Low	Low	0	25	406	0	2
16	17	F	Low	Low	2	268	362	0	4
17	18	M	Low	-	2	128	208	-	-
18	35	M	Low	-	0	126	264	-	-
19	49	F	High	Low	0	147	1322	1,5	3
20	53	M	High	Low	0	14	1217	2	8
21	77	F	High	High	0	25	2674	0	13
22	45	F	Low	Low	0	25	462	0,5	4,5
23	24	F	Low	Low	0	45	361	0	6
24	55	M	Low	Low	0	65	462	0,5	2
25	21	M	High	High	19	221	838	4	7
26	68	M	Low	Low	0	72	164	0	1,4
27	61	M	High	High	8	336	1480	7	8,5
28	63	M	Low	Low	0	106	196	0,5	4,5
29	41	F	Low	Low	0	9	731	0	1
30	69	M	High	High	22	237	574	3	4,4
31	43	M	High	High	19	238	218	4	10
32	39	F	High	Low	10	274	469	6	5
33	43	F	Low	High	0	7,8	699	0	18
34	63	F	High	High	0	17,3	1800	0	28
35	44	F	Low	Low	0	141	543	1,5	3,4
36	75	M	High	Low	8	590	230	6,3	3

Abbreviations: M/F, male/female; WBC, white blood cell count; PB, peripheral blood.

1 Age at diagnosis.

2 Measured in cm below the left costal margin as assessed by palpation.

### CD34 separation and Bcr/Abl fluorescent in situ hybridization

CD34 positive cells from five CML patients were sorted from peripheral blood as described in [24]. fluorescent in situ hybridization (FISH) was run on the samples as described in [24].

### Antibodies and staining for flow cytometry

Antibodies used for extracellular staining were α-CD3-FITC (fluorescein isothiocyanate), α-CD3-APC (allophycocyanin), α-CD4-FITC, α-CD8-PE (phycoerytrin), α-CD8-FITC, α-CD11b-PE/Cy5, α-CD14-FITC, α-CD33-PE, α-CD34-APC, α-PD-1-FITC, α-PD-L1-PE (clone: 29E.1A3), IgG1 κ-APC, IgG1 κ-FITC, IgG2b κ-PE, IgG2a κ-PE/Cy5 all from Biolegend (San Jose, CA, USA). For blocking in cell culture experiments α-PD-L1 antibody (clone: 29E.2A3, Biolegend) or isotype control (clone: MPC 11, Biolegend) were used. Stainings for flow cytometry were made on patient leukapheresis (from patients 1–18 in Table 1) and control samples. The samples were thawed in PBS and run through a MACS pre-separation filter (Miltenyi Biotech, Bergisch Gladbach, Germany) to remove clumps of dead cells. Unspecific antibody binding was blocked with 1% bovine serum albumin (BSA) (Sigma Aldrich, St Louis, MO, USA) in PBS and cells were stained for different surface markers. For staining of MDSCs (patients 1–9 and 11–18 in Table 1), cells were stained for the surface markers CD34, CD11b, CD14, and CD33. For detection of PD-L1 on tumor cells the cells (patients 1–12 and 14–18 in Table 1) were stained for CD34, CD11b, and PD-L1. The expression of PD-1 on T cells (patients 1–18 in Table 1) was analyzed by staining of CD3, CD8, and PD-1. Isotype controls were used to exclude unspecific binding from the analysis. Cells were analyzed on LSRII (BD Biosciences, Franklin Lakes, NJ, USA) and the data were evaluated with Flow Jo (Tree star, Ashland, OR, USA) and BD FACSDiva (BD Biosciences) software. At analysis live cells were gated depending on the FSC and SSC properties of the cells. For gating strategies, see Figures S2, S3, S4 and S6. Median fluorescence intensity (MFI) of PD-L1 was calculated by subtracting the MFI of the isotype control from the MFI of the sample. For PD-1 the MFIs of the isotype controls from both patients and HCs were at the same level, hence MFIs of the samples were reported without subtraction of isotype MFIs.

### RNA isolation and cDNA synthesis

Cryopreserved blood samples from CML-patients (n = 6, patients 11, 24–27 and 32 in Table 1) and control subjects (n = 9) where red blood cells had been removed were thawed and run through a MACS pre-separation filter (Miltenyi Biotech) to remove clumps of dead cells. For total RNA isolation, RNeasy Mini Kit (Qiagen, Hilden, Germany) was used and the isolation was made according to the manufacturer's instructions. Also RNA from CML cell lines K562, BV-173 and CML-T1 was isolated using the same kit. To remove possible DNA contamination, a DNase free set (Qiagen) was used as instructed by the manufacturer. cDNA was synthesized from up to 0,5 µg RNA with iScript cDNA synthesis kit according to the manufacturer's instructions (BioRad, Hercules, CA, USA).

### Arginase 1 real time PCR

Real time PCR was performed on cDNA prepared from CML cell lines and leukocytes from CML patients (n = 6, patients 11, 24–27 and 32 in Table 1) and control subjects (n = 9) with the CFX96 Real-Time System (BioRad). Reactions were preformed with the SYBR Green Supermix (BioRad). Gene specific primers were used for Arg 1: 5′-GTT TCT CAA GCA GAC CAG CC-3′ (Fw), 5′-GCT CAA GTG CAG CAA AGA GA-3′ (Rv), for b-actin 5′-CGA GAA GAT GAC CCA GAT CAT G-3′ (Fw), 5′- ACA GCC TGG ATA GCA ACG TAC A-3′ (Rv). The protocol for the reaction was one cycle at 95°C for 3 min, and 40 cycles at 95°C for 9 seconds followed by 60°C for 1 min. Amplification steps were followed by a melting curve. Data were evaluated using the BioRad CFX Manager Software (BioRad) and Arg1 expression was normalized to β-actin expression.

### T cell proliferation assay with programmed death receptor ligand 1 blockade – cell lines

50 000 PBMCs from healthy donors were plated in a 96 well plate and stimulated with 80 IU/ml IL-2 (Proleukein, Novartis, Basel, Switzerland). Cells from the two cell lines K562 and BV-173 were irradiated at the dosage of 60 Gray (Gy) and 12 Gy respectively and blocked with 5 µg/ml PD-L1 blocking antibody (Biolegend), or 5 µg/ml isotype control (Biolegend) before being added to the PBMCs. Blocked cell lines were then mixed with PBMCs in a 1∶1 ratio and co-cultured for 2 days in RPMI-1640 media supplemented with 10% FBS and 1% PEST. As controls, cell lines and PBMCs were cultured alone. After 2 days thymidine ^3^H (PerkinElmer, Waltham, MA, USA) was added and cells were harvested (Harvester 96® Mach III M, Tomtec, Hamden, CT, USA) on to a membrane after 6 hour of incubation. Incorporation of thymidine ^3^H was measured by the 1450 Microbeta® TriLux from PerkinElmer. Proliferation of PBMCs was evaluated by subtracting the amount of thymidine ^3^H incorporated in the cell lines cultured alone from the incorporation of thymidine ^3^H in the PBMC/cell line co-cultures.

### T cell proliferation assay with programmed death receptor ligand 1 blockade and in vitro T cell stimulation – patient samples

Cryopreserved CML samples from peripheral blood (n = 11, patient 11, 19–27 and 32 in Table 1) and control samples from buffy coats (n = 10) were thawed and cultured in RPMI-1640 media supplemented with 10% FBS and 1% PEST over night. PBMCs from one healthy donor were thawed and cultured over night. The next day CD3 positive MACS microbead selection was run on patient and control subject samples, according to the manufacturer's instructions. Fc-recptor block was added to the cells before the addition of microbeads. All reagents were from Miltenyi Biotech. After selection, CD3 negative cells were cultured in RPMI-1640 media supplemented with 10% FBS and 1% PEST for 2–3 hours before being used in proliferation assays. After 2–3 hours CD3 negative cells from patients and control subjects were divided into two tubes per sample, pelleted and incubated with 3 µg/ml PD-L1 blocking antibody or isotype control, respectively, for 30 minutes. Blocked cells were mixed with responder PBMCs from a control subject in a 1∶1 ratio. Cells were pelleted and resuspended in RPMI 1640 media supplemented with 10% FBS and 1% PEST and plated in 96-well plates. PD-L1 blocking antibody or isotype control (3 µg/ml), respectively, was added to the wells together with 100 IU/ml IL-2 (Novartis). For *in vitro* stimulation of T cells CD3 cells were cultured in 96-well plates with or without the addition of 1,7 µg/ml anti CD3-antibody (clone OKT-3, BioLegend) and 100 IU/ml IL-2. After 2 days of culture, proliferation in co-cultures and *in vitro* stimulated CD3 cells was evaluated using Click it proliferation kit (Invitrogen) according to the manufacturer's instruction. Briefly, 5 µM 5-ethynyl-2′-deoxyuridine (EdU) was added to proliferation assays and cells were cultured over night to incorporate EdU into DNA of proliferating cells. Cells were washed and to exclude dead cells from analysis, the LIVE/DEAD staining kit (Invitrogen) was used according to the manufacturer's instruction. Cells were blocked in 1% BSA where after T cells were stained with a cocktail of α-CD3-FITC, α-CD4-FITC and α-CD8-FITC. EdU incorporation was detected with Click it detection reagent mix. The reaction was performed in smaller volume than stated in protocol from manufacturer and the components of the reaction mixture were scaled down accordingly. Samples were analyzed on LSRII (BD Bioscience) and data were analyzed with Flow Jo software (Tree Star). Supernatants from co-cultures (patients 11, 19–27 and 32 from Table 1) were taken for IL-2 ELISA after overnight incubation with EdU and stored in −80° until analysis.

### Arginase 1, soluble CD25 and IL-2 ELISA

Arg 1 protein levels in plasma from CML patients (n = 10, patients 11, 19–20, 25, 29 and 32–36 in Table 1) were assessed using an ELISA from Hycult Biotech (Plymouth Meeting, PA, USA). The ELISA was performed according to the manufacturer's instructions. Arg 1 can be released from lysed erythrocytes, hence to avoid false positive values hemolysed plasma samples were excluded from the analysis. The level of sCD25 in plasma from CML patients (n = 14, patients 11 and 20–32 in Table 1) and control subjects (n = 18, control subjects plasma samples were a subpopulation derived from a cohort of healthy donors previously published in [21]) was measured using a sCD25 ELISA from Diaclone (Besancon Cedex, France) according to the manufacturer's instructions. To determine the IL-2 concentration in supernatants from proliferation experiments, an ELISA using IL-2 Eli-Pair set (Diaclone) was performed.

### Statistical analysis

Statistical analyses were made with Prism Software (Graphpad Software Inc., La Jolla, CA, USA). To assess statistical significance of difference between more than two groups the Kruskal Wallis test was used. When statistical significant difference between groups was found, Dunn's post test was applied.

## Results

### Patient characteristics

All CML patients were newly diagnosed and in chronic phase. The mean age of the patients was 46 years ranging from 17 to 77 years. Twenty one patients were Sokal high risk (HR) and 15 were Sokal low risk (LR) (Table 1). Eight patients (patient 3, 5, 21, 22, 25, 28, 31 and 34 in Table 1) had been treated with hydroxyurea for 1–4 days prior to leukapheresis or blood sampling, patients were otherwise untreated. After the initiation of this study, a new scoring system for CML patients on TKI treatment, called the EUTOS score [25], has come to light. EUTOS score of the patients can be seen in Table 1, but all analyses compare the Sokal HR and LR groups.

### CD34 positive cells from peripheral blood of chronic myeloid leukemia patients originates from the leukemic clone

Peripheral blood from five CML patients was sorted with magnetic beads and Ph positivity in CD34 positive cells was measured by FISH. The percentage of FISH positive cells in the CD34 fraction ranged from 77–98% with a median of 95% (see Figure S1)

### Chronic myeloid leukemia patients have increased myeloid derived suppressor cell numbers and high arginase 1 levels

**Figure 1:**
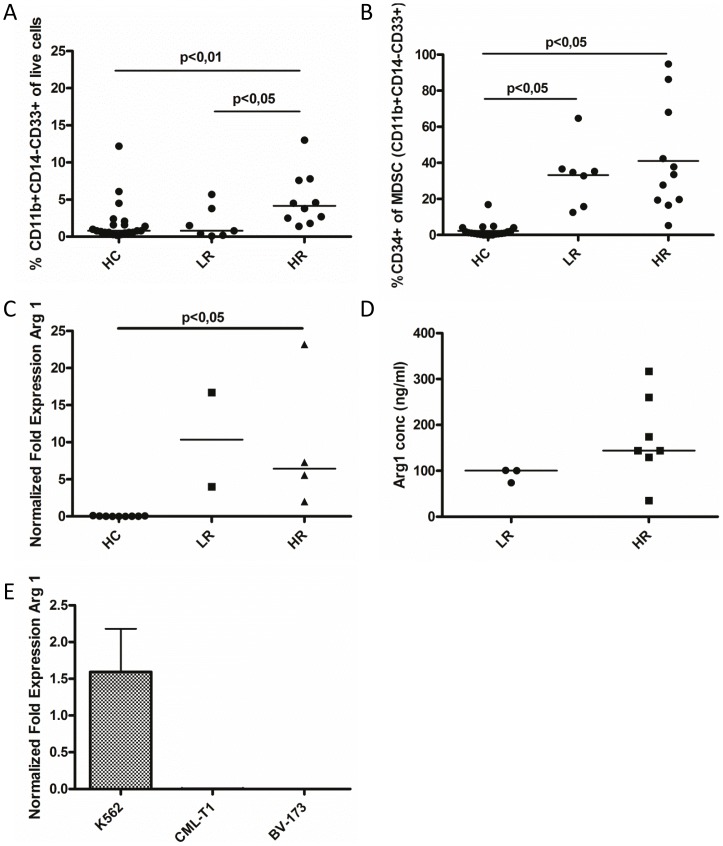
Myeloid derived suppressor cell (MDSC) and arginase 1 (Arg1) levels. (A) Levels of MDSCs (CD11b+CD14−CD33+) in different CML patient risk groups (high risk (HR) n = 10, low risk (LR) n = 7) and healthy control subjects (HC, n = 21) as shown by flow cytometry. (B) Levels of MDSCs expressing CD34 in CML patient risk groups (HR n = 10, LR n = 7) and HCs (n = 21). (C) Arg1 mRNA expression in HCs (n = 9) and CML patients (HR n = 4, LR n = 2) assessed by real time PCR. Statistically significant differences between groups are indicated by P-values in the figures. (D) Arg1 concentration in CML patient plasma (HR n = 7, LR n = 3) measured by ELISA. (E) Arg 1 mRNA expression in CML cell lines. The plot shows mean values with standard error of the mean.

The level of MDSCs, here defined as CD11b+CD14−CD33+ cells, was investigated using flow cytometry (for gating strategy, see Figure S2). We found that MDSC levels were similar in the whole CML patient population (n = 17) compared to control subjects (n = 21). However, when dividing patients into HR (n = 10) and LR (n = 7) groups, the HR group had statistically significant higher MDSC level compared to both LR patients and control subjects (p<0,05 and p<0,01 respectively, Figure 1A). Since MDSCs and CML tumor cells share some features we hypothesize that the tumor cells themselves may be MDSCs. At the time of the study there were no specific markers for CML tumor cells available for flow cytometry analysis. However, since we show that 95% of CD34 positive cells in peripheral blood were Ph positive (Figure S1) CD34 was used as a marker for CML tumor cells. When investigating CD34 expression on CML patient MDSCs we found that a median of 35% of MDSCs expressed CD34 demonstrating that some tumor cells may be accounted for as MDSCs (Figure 1B). Investigating CML cell lines K562 and BV-173 for MDSC markers, we found that in both cell lines about half of the cells expressed the MDSC markers (data not shown). The expression of the MDSC-associated molecule Arg1 was assessed in leukocytes from CML patients (HR n = 4, LR n = 2) and control subjects (n = 9) as well as in CML cell lines with real time PCR. The relative expression of Arg1 in leukocytes was higher in HR patients compared to control subjects (p<0,05, Figure 1C) Statistical difference between control subjects and LR patients could not be determined because of too few LR samples. Arg1 mRNA levels were also determined in four more patients that were in accelerated phase or Sokal intermediate risk. Also in these patients Arg1 mRNA levels were higher than in HCs (data not shown). Arg1 protein levels in CML patient plasma (LR n = 3, HR n = 7) were assessed with ELISA. In all patients the Arg1 plasma levels, ranging from 35–316 ng/ml (Figure 1D), were higher than reported normal median levels (9.56±4.03 ng/mL to 21.9±9.2 [26], [27], [28]).The CML cell line K562, but not the cell lines BV-173 or CML-T1, expressed Arg1 mRNA (Figure 1E).

### Chronic myeloid leukemia cells express high levels of the co-inhibitory molecule programmed death receptor ligand 1

**Figure 2:**
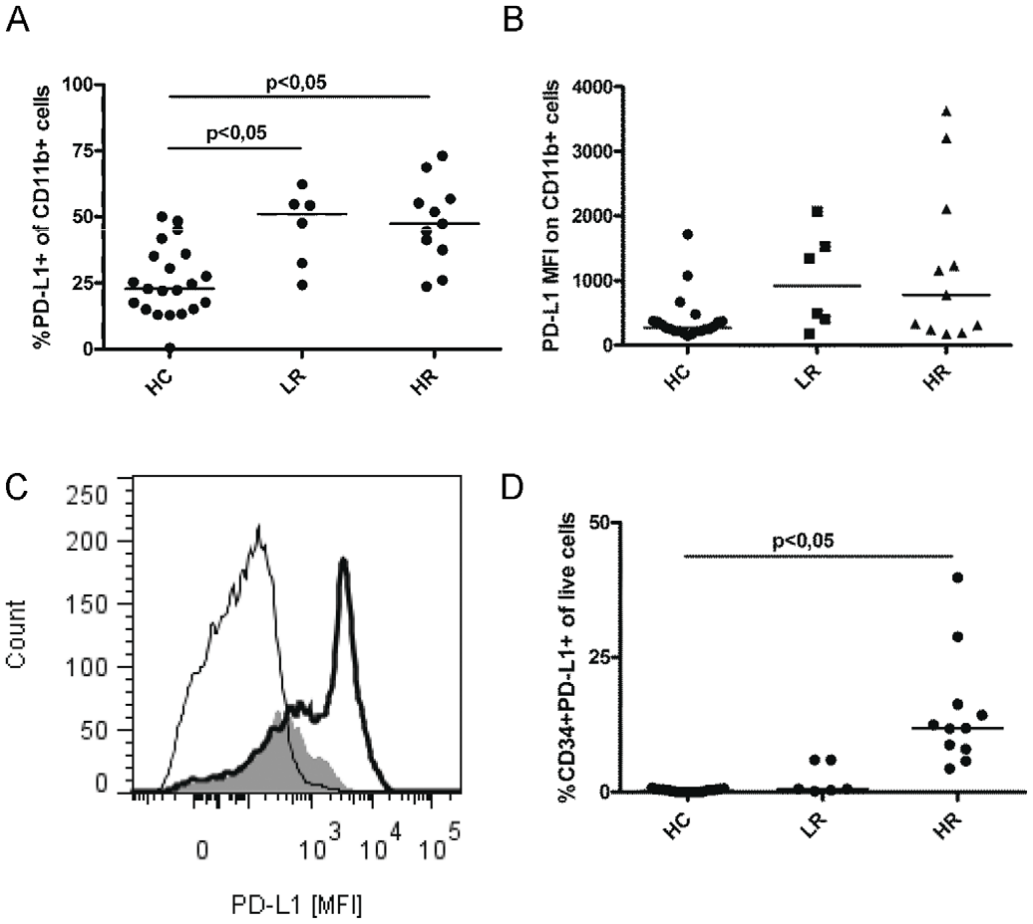
Programmed death receptor ligand 1 (PD-L1) expression levels on patient cells. (A) PD-L1 expression on myeloid (CD11b+) cells from patients (HR n = 11, LR n = 6) and HCs (n = 21) as assessed by flow cytometry. (B) Median fluorescence intensity (MFI) of PD-L1 on CD11b cells. (C) MFI of PD-L1 expression on CD11b cells from a representative healthy control (filled histogram) and CML patient (thick line), the thin line represents the isotype control. (C) Percent PD-L1 expressing CD34 cells of all live cells in HCs (n = 21), LR (n = 6) and HR (n = 11) patients. Statistically significant differences between groups are reported as P-values in the figures.

Myeloid cells (CD11b+) in CML patients and control subjects were analyzed for the co-inhibitory molecule PD-L1 expression by flow cytometry (for gating, see Figure S3). A median of 48% of CD11b cells from CML patients (n = 17) expressed PD-L1, this was significant different from the expression in control subjects (n = 21) where 23% of CD11b cells expressed PD-L1 (p<0,05). There was no difference between LR or HR groups concerning the percentage PD-L1 expressing CD11b cells (Figure 2A). The expression level of PD-L1 molecule on single CD11b cells, assessed as median fluorescence intensity (MFI) was not significantly higher in patients compared to control subjects (Figure 2B). In most patients however, there was a subgroup of CD11b cells expressing high levels of PD-L1, this subgroup was not seen as frequently in control subject cells (Figure 2C and Figure S3). HR patients had a significantly increased level of PD-L1 expressing CD34 cells compared to control subjects (p<0,05) while LR patients resembled the control subjects (Figure 2D). However, most PD-L1 positive cells were found in the CD11b+CD34− population in HR patients. The same trend was seen in LR patients but the difference did not reach significance (data not shown). Overall, PD-L1 positive peripheral leukocytes were more frequent in CML patients than in control subjects (data not shown).

### T cells from chronic myeloid leukemia patients express high levels of programmed death receptor 1

**Figure 3:**
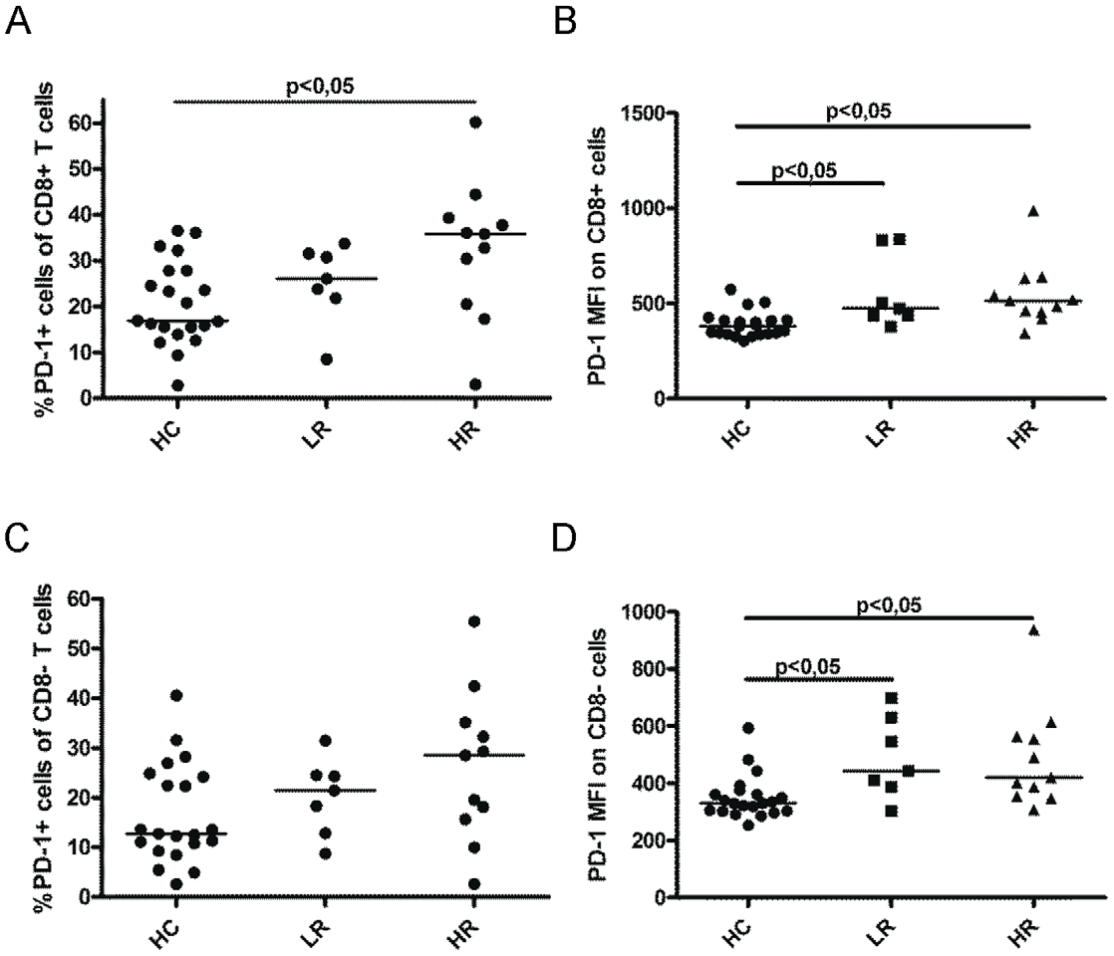
Programmed death receptor 1 (PD-1) expression on T cells. (A) The percentage of PD-1 positive cytotoxic T cells (CD3+CD8+) in HCs (n = 21), LR (n = 7) and HR (n = 11) as determined by flow cytometry. (B) The PD-1 MFI is shown for cytotoxic T cells. (C) The percentage of PD-1 positive T helper cells (CD3+CD8−) in HCs (n = 21), LR (n = 7) and HR (n = 11) patients was determined by flow cytometry. (D) The PD-1 MFI for helper T cells. Statistically significant differences between groups are indicated as P-value in the figure.

To investigate the relevance of PD-L1 expression on myeloid cells, the expression of its receptor PD-1 was analyzed on patients' and control subjects' T cells (for gating strategy, see Figure S4). The HR patients (n = 11) had a significant increase of PD-1 positive CD3+CD8+ cytotoxic T cells compared to control subjects (n = 21) (p<0,05, Figure 3A). Moreover, the PD-1 expression on the cell surface of CD8 cells was higher in both LR and HR patients compared to control subjects (p<0,05 and p<0,05 respectively, Figure 3B and Figure S4). There was a slightly higher level of CD8 positive and a slightly lower level of CD8 negative T cells in HR patients compared to healthy controls (Figure S5). T helper cells (analyzed as CD3+CD8− cells) expressing PD-1 seemed to be increased in HR patients (n = 11) compared to control subjects (n = 21) but the difference did not reach significance (Figure 3C). However, there was an increase in PD-1 MFI on CD8 negative cells both in LR and HR patients compared to controls (p<0,05 and p<0,05 respectively, Figure 3D).

### The effect of programmed death receptor ligand 1 blockade

**Figure 4:**
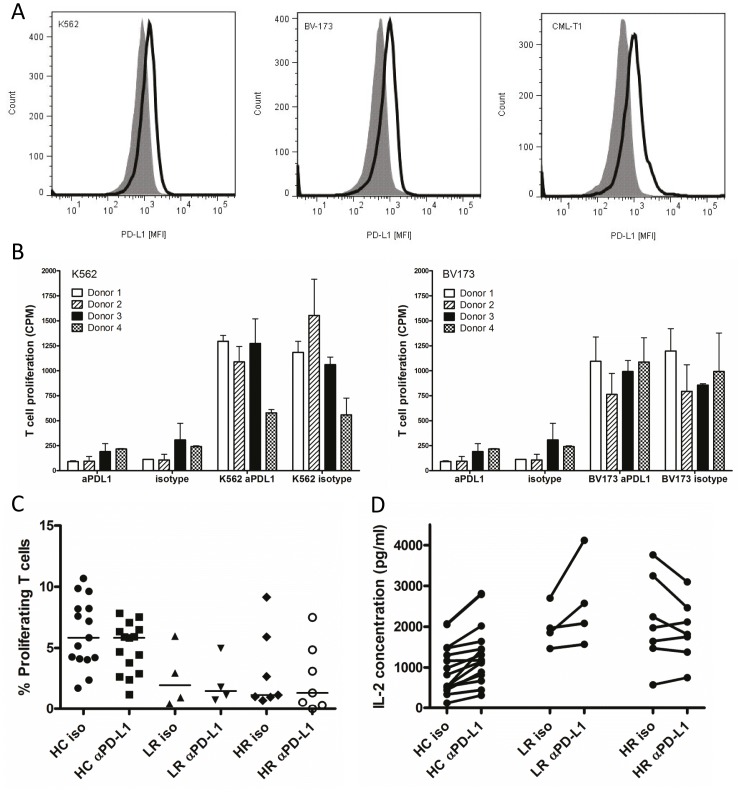
Blocking of programmed death receptor ligand 1 (PD-L1) in co-cultures. (A) PD-L1 expression on the CML cell lines K562, BV-173 and CML-T1 was evaluated by flow cytometry. The thick line represents staining with specific antibody and the filled histograms represent the staining with isotype control antibody. (B) Proliferative response of healthy donor PBMCs to CML cell lines with or without addition of PD-L1 blocking antibody. The bars show the proliferative response in different wells after subtraction of proliferation seen when cells lines were cultured alone. The experiment was repeated twice with four donors. Proliferation was measured by thymidine H^3^ incorporation on triplicate samples. (C) T cell-depleted leukocytes from CML patients (HR n = 7, LR n = 4)) and HCs (n = 15) were co-cultured with PBMCs from a control subject (responder) with addition of an irrelevant antibody (iso) or an antibody blocking PD-L1 (αPD-L1). The percentage of proliferating T cells in the co-culture is shown. (D) The concentration of IL-2 in co-culture supernatants from HCs (n = 15), LR (n = 4) and HR (n = 7) was assessed by ELISA. The increase/decrease in IL-2 concentration for the individual control subjects and CML patients are shown in the figure.

CML cell lines K562, CML-T1 and BV-173 were all positive for PD-L1 expression as analyzed flow cytometry (Figure 4A). K562 and BV-173 were used in a proliferation assay to investigate if PD-L1 blockade would increase the proliferative response of healthy donor PBMCs. All donors reacted to the tumor cell lines by proliferation. However, the effect of PD-L1 blockade was modest implicating that PD-L1-induced tolerance is not of great importance in this setting (Figure 4B). PD-L1 expression is also seen on CML patient cells as seen in Figure 2A. To investigate if the PD-L1 expression on CML patient cells can inhibit proliferation of healthy T cells and if this inhibition could be released by PD-L1 blockade, PBMCs from an individual control subject (responder cells) were stimulated with IL-2 and co-cultured with CD3 negative CML cells (HR n = 7, LR n = 4) or cells from control subjects (n = 15) (inhibitor cells) (for gating strategy, see Figure S6). CD3 negative cells were chosen as inhibitor cells to include as many PD-L1 expressing cells as possible. There was no significant difference in T cell proliferation in cultures with CML cells from HR or LR patients (Figure 4C). Blocking PD-L1 on the inhibitor cells by adding α-PD-L1 antibodies did not affect T cell proliferation (Figure 4C). To assess T cell function and the inhibition thereof in another manner, the IL-2 concentration in the culture supernatants was evaluated. There was a slight, increased in IL-2 concentration after PD-L1 blockade in samples from most control subjects and LR patients while a majority of samples from HR patients had a slight IL-2 concentration decrease after blockade (Figure 4D). This proliferation experiment was also run with cells from another four CML patients that were either Sokal intermediate risk patients or accelerated phase patient similar results in proliferation and IL-2 secretion was seen for these patients (data not shown).

### T cell proliferation after in vitro stimulation is not altered in CML

Proliferative response from sorted T cells from CML patients and control subjects was assessed after *in vitro* stimulation (HC n = 10, HR n = 3, LR n = 3). There was no difference in proliferation of stimulated cells from CML patients and healthy controls (Figure S7).

### Soluble CD25 plasma levels are increased in chronic myeloid leukemia patients

**Figure 5:**
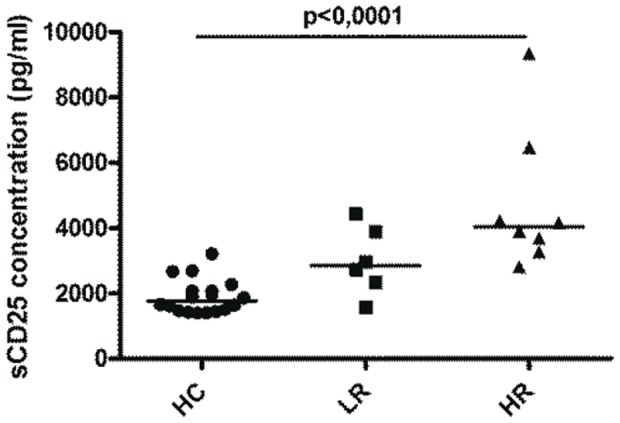
Soluble CD25 concentration. The concentration of soluble CD25 in blood plasma from CML patients (LR n = 6, HR n = 9) and HCs (n = 18) was investigated with ELISA. The figure shows soluble CD25 concentration expressed as pg/ml in blood plasma. Statistically significant difference between groups is reported as P-value in the figure.

Another mechanism of T cell suppression is the deprivation of IL-2 and/or other important factors for T cell growth. We have previously shown that sCD25 can suppress T cell proliferation likely by sequestering IL-2. Therefore we sought to investigate the presence of sCD25 in CML patients. The level of sCD25 in blood plasma from CML patients (n = 14) and control subjects (n = 18) was studied using ELISA. The level was significantly higher in HR patients compared to the level in control subjects (p<0,0001 Figure 5).

## Discussion

The impact of the immune system and the importance of immune escape mechanisms in cancer development have been shown for various cancer forms including hematologic malignancies [29], [30]. In this study we have investigated the presence of different immune escape mechanisms in CML. The impact of the immune response in hematologic malignancies such as CML is especially intriguing since the malignant transformations occur in cells of the immune system. Furthermore in the only proven curative treatment of CML, allogeneic stem cell transplantation, the graft versus leukemia effect is thought to be of importance implicating the importance of immune control in CML. To better understand the immune status in CML and to compare the immune status of HR and LR patients, samples from newly diagnosed patients were investigated for the presence of MDSCs, Arg-1, PD-L1/PD-1, as well as sCD25, all important regulators of T cell anti-tumor immunity. Since one of our goals was to compare HR and LR patients we aimed to investigate the same amount of LR and HR leukapheresis samples. However, since fewer LR patients undergo leukapheresis fewer LR samples could be investigated in our study.

Recruitment of MDSCs is a known immune escape mechanism exerted by many solid tumors [11], [31], [32] and the level of circulating MDSCs have been correlated with disease stage in breast cancer [33]. MDSCs are characterized as immature myeloid cells and since CML cells mostly consist of immature myeloid cells and thus share features with MDSCs, we hypothesized that the tumor cells themselves might be MDSCs. Supporting this hypothesis are our results showing that two CML cell lines tested both expressed MDSCs markers. Furthermore, we show that most patients had CD34 cells (mostly tumor cells) expressing MDSC markers. However, the majority of MDSCs did not express CD34. In our studies we define MDSCs as CD11b+CD14−CD33+ cells, a phenotype that others have shown to be suppressive in different cancer forms like renal cell carcinoma and soft tissue sarcoma [34]. In our cohort of CML patients we could, in peripheral blood, see an increased level of MDSCs in HR patients compared to control subjects. In some patients with solid cancers high levels of MDSCs correlate with a worse prognosis [29]. Interestingly, in our study HR patients had higher levels of MDSCs compared to LR patients, hence the correlation with worse prognosis may be true also for CML, but it needs to be confirmed in a larger study. We have also shown that CML cells from peripheral blood have higher expression of Arg1 than cells from healthy control subjects and that our CML cohort had high Arg1 plasma levels. This is in concordance with results from other groups showing high levels of Arg1 in cancer patient plasma or serum [29], [35]. Since expression of Arg1 can result in inhibition of T cells [17] the increased levels of MDSCs and high expression level of Arg1 in CML patients may promote immune escape in CML.

Next, we investigated the presence of the immune regulatory molecules PD-1 and PD-L1. PD-L1 is normally expressed on different immune cells like T cells, B cells, dendritic cells and macrophages as well as non-immune cells like endothelial cells and pancreatic islets and functions as an important immune regulatory mechanism as it inhibits T cells by binding to PD-1 upregulated on activated T cells [36]. Besides being an important immunoregulatory mechanism the PD-1/PD-L1 interaction is also an important immune escape mechanism in some cancers and infectious diseases. This is illustrated by the fact that antibodies blocking the interaction can induce remission in patients with advance stage solid tumors [37], [38], [39], [40]. Moreover, PD-L1 on tumor cells and PD-1 expression on tumor-infiltrating cells has been reported to correlate with advanced stage disease and a worse prognosis by some investigators [30], [41], [42] while others have found no correlation between PD-L1 expression on tumor cells and the prognosis of the patients [43]. Since CML cells originate from immune cells it is likely that they can play a part in modulating the immune responses for example by high expression of PD-L1. In our study we have shown that CML patients expressed higher levels of PD-L1 on myeloid cells compared to control subject cells. In concordance with Mumprecht et al we also show that the expression of PD-1 was higher on cytotoxic T cells from CML patients than those from control subjects [20]. In a CML mouse model these authors showed that blocking the PD-1/PD-L1 interaction increased the survival of CML mice in blast crisis indicating that PD-1/PD-L1 interaction might be an important immune inhibitory mechanism in CML [20]. To investigate the contribution of PD-1/PD-L1 interaction to immune regulation in our CML cohort, the proliferation of healthy T cells in response to CML patient cells and cell lines in the presence of PD-L1 blocking antibodies was studied. We found that blocking the PD1/PD-L1 interaction did not increase proliferation of healthy T cells. These results are in line with those from Salih showing that PD-L1 blockade did not induce T cell activation despite high PD-L1 expression on patient cells and leukemic cell lines co-cultured with the T cells [44]. Taken together these results implicate that PD-1/PD-L1 interaction may have different functions in solid tumors and in leukemia. Furthermore, results from mouse models may not always translate into the human setting. Moreover, our data are obtained *in vitro* and may not correlate to the effect *in vivo*. It is also important to consider that our co-culture experiment illustrated the ability of T cells to proliferate and not the T cell function. PD-L1 interaction with PD-1 on T cells leads not only to inhibition of proliferation but also to decreased IL-2 secretion by blocking downstream pathways of PI-3 kinase and Akt [45]. Therefore, to further investigate the influence of PD-L1 blockade on T cell function we measured the IL-2 concentration in co-culture supernatants. PD-L1 blockade increased the concentration of IL-2 in the medium from most healthy control samples and in LR patients indicating that the interruption of PD-1/PD-L1 interaction enhanced T cell function. However, in co-cultures with CML cells from most HR patients the IL-2 concentration in the medium was decreased after addition of blocking antibody implicating that the PD-1/PD-L1 interaction may have different function in these patients. Clearly, the PD-L1/PD-1 pathway in leukemia needs further investigation before testing PD1/PD-L1 blockade as treatment for CML.

The lack of increased *in vitro* T cell proliferation after PD-L1 blockade may reflect that there are immune escape mechanisms other than PD-L1 blocking T cell responses in CML. Moreover, in *in vitro* stimulation T cell proliferation assays we saw no inhibition of CML T cell proliferation compared to T cells from control subjects which may implicate that T cell suppression does not occur when T cells are separated from the suppressive tumor cells. One of these mechanisms could be the increased concentration of sCD25 that we have detected in CML blood plasma. sCD25 can act immunosuppressive by binding free IL-2 [46], thereby inhibiting it to bind to and support T cell activation. We as well as others have shown that patients with hematological malignancies, such as B cell malignancies and CML, have increased level of sCD25 in plasma [21], [23], [47], [48]. Several studies have shown that patients in advanced stages of disease have higher levels of sCD25 in plasma compared to patients in less advance stages and that sCD25 can be a prognostic factor for patients with lymphoma [47], [49], [50], [51], [52]. In CML, patients in accelerated phase and blast crisis have higher levels of sCD25 in plasma [47], [50]. In the present paper we show that the level of sCD25 in plasma is higher in HR patients. Since high levels of sCD25 have been associated with activated T cells [22] this could indicate many activated T cells in CML patients, especially in HR patients. However, our other results presented here do not indicate a high level of T cell activation, thus, the high level of sCD25 probably originates from another source. Since HR patients have higher tumor burden and also higher sCD25 levels compared to LR patients we hypothesize that the sCD25 might be secreted from the tumor cells. This hypothesis is in line with results from Motoi et al that show that the level of sCD25 in plasma is correlated with the amount of blasts and leukocytes in peripheral blood of CML patients in blast crisis [48]. Moreover, Hermann et al have shown that leukemic stem cells from CML patients express CD25 on their surface further supporting that CD25 may be produced and perhaps released from CML tumor cells [53].

Taken together, anti-tumor reactive T cells in CML may be controlled by multiple immune escape strategies including recruitment of MDSCs, expression of Arg1, PD-L1, PD-1, and sCD25. Some of these immune escape mechanisms seem to be more pronounced in some Sokal HR patients, if the high prognostic score is a consequence of immune escape mechanisms in the patients or if the high prognostic score (and probably a higher tumor burden) leads to immune escape mechanisms needs to be further investigated. Previously it has been shown that CML patients with a higher tumor burden had lower levels of T cells directed towards the junction of the Bcr/Abl protein compared to patients with lower tumor burden [54] implicating that the Bcr/Abl junction is immunogenic and that a low tumor burden can stimulate an immune response while high tumor burden on the other hand may suppress T cells, possibly by membrane bound or secreted factors. For some of the parameters studied in this paper, the significant difference seen between HR patients and control subjects in expression levels is created by a few outliers. These patients may be of importance to study since they may be patients that are less susceptible to immunotherapy, these may also be the patients that relapse after stopping TKI treatment. It has been shown that HR patients have an increased risk of relapse after TKI treatment cessation [6]. The presence of immune escape mechanisms could aggravate the use of immunotherapy such as IFNα in CML and should therefore be monitored during such studies. This is especially relevant for upcoming trials combining IFNα and TKIs since IFNα is added to improve the anti-tumor response. Furthermore, the effect of TKIs on immune escape mechanisms in CML needs to be studied since TKIs has been shown to have effect on other immune cells.

## Supporting Information

Figure S1 **Level of Ph+ cells.** The level of Ph+ cells was measured by FISH in sorted CD34 cells from peripheral blood from CML patients.
(TIF)

Figure S2 **Stategy for MDSCs gating.** The percentage MDSCs of live cells were calculated as the percentage of cells inside the “live” gate that were CD11b+CD14−CD33+. The percentage CD34 cells of MDSCs were calculated as the percentage CD34 positive cells inside the MDSC gate.
(TIF)

Figure S3 **Strategy for gating CD11b and CD34 cells expressing PD-L1.** The percentage of PD-L1 positive cells in the CD11b population was calculated as the percentage of PD-L1 positive cells inside the CD11b gate. PD-L1 MFI histograms were created from the CD11b gate, isotype control is shown as a thin line. The percentage of CD34+PD-L1+ of live cells was calculated as the percentage CD34+PD-L1+ cells in the “live” gate.
(TIF)

Figure S4 **Strategy for gating PD-1 positive T cells.** The percentage of PD-1 cells of CD8 cells was calculated as the percentage of PD-1 positive cells (right upper quadrant) of total CD8 cells (left and right upper quadrants). The percentage of PD-1 cells of CD8 negative cells was calculated as the percentage of PD1 positive cells (right lower quadrant) of total CD8 negative cells (left and right lower quadrant). PD-1 MFI histograms were created from CD8 cell gate (left and right upper quadrants), isotype control is shown as a thin line.
(TIF)

Figure S5 **T cell levels.** Level of CD8 (A) and CD8 negative (B) cells in LR (n = 7) and HR (n = 12) patients compared to HR (n = 21). Statistically significant differences between groups, reported as P-value in the figures were assessed by the non-parametric Kruskal Wallis test and Dunn's post test.
(TIF)

Figure S6 **Strategy for gating proliferating T cells.** The proliferating cells were calculated as the percentage of cells positive for the proliferation marker EdU in the CD3 gate.
(TIF)

Figure S7 **Proliferation of** ***in vitro*** **stimulated T cells.** The percentage of proliferating T cells after *in vitro* stimulation of T cells from HCs (n = 10), LR (n = 3), HR (n = 3) patients.
(TIF)
