# Supplementary figures and images for: Increased Level of Myeloid-Derived Suppressor Cells, Programmed Death Receptor Ligand 1/Programmed Death Receptor 1, and Soluble CD25 in Sokal High Risk Chronic Myeloid Leukemia
Source: PLoS One. 2013 Jan 31;8(1):e55818. doi: 10.1371/journal.pone.0055818 (PMC3561335; doi:10.1371/journal.pone.0055818)

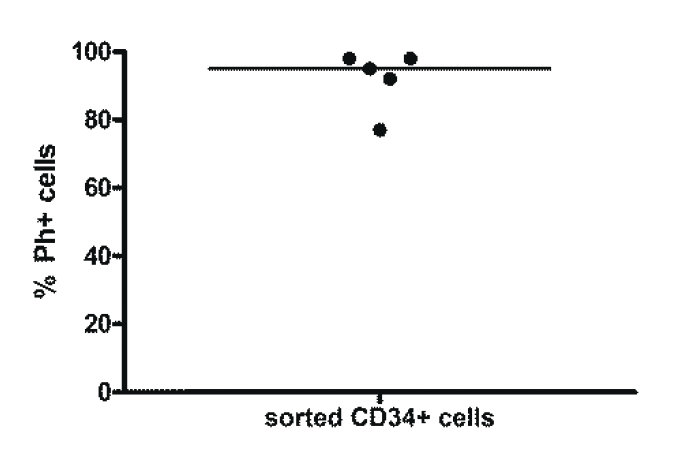

Supplement: Figure S1 — Level of Ph+ cells. The level of Ph+ cells was measured by FISH in sorted CD34 cells from peripheral blood from CML patients. (TIF) [file pone.0055818.s001.tif]

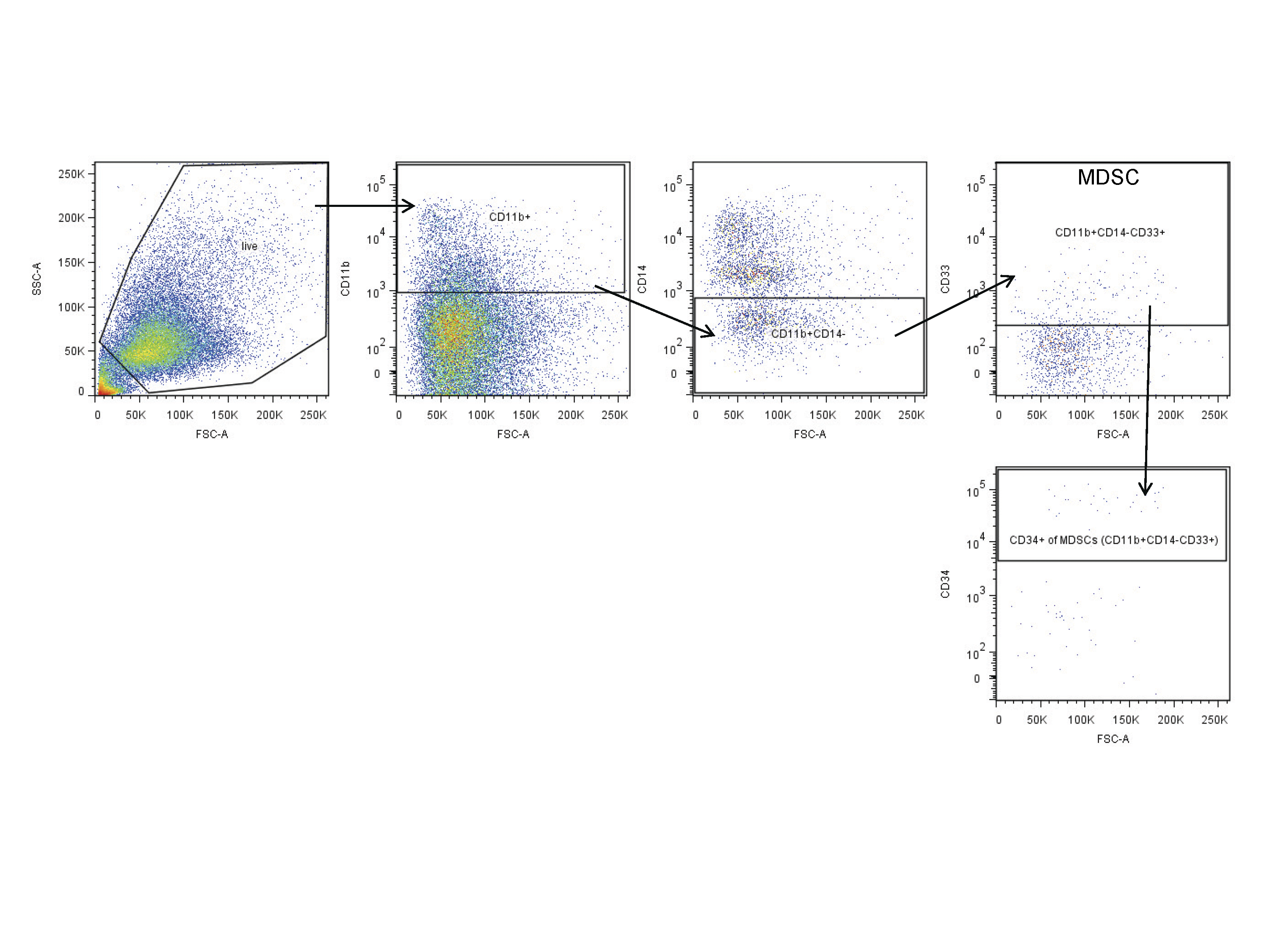

Supplement: Figure S2 — Stategy for MDSCs gating. The percentage MDSCs of live cells were calculated as the percentage of cells inside the “live” gate that were CD11b+CD14−CD33+. The percentage CD34 cells of MDSCs were calculated as the percentage CD34 positive cells inside the MDSC gate. (TIF) [file pone.0055818.s002.tif]

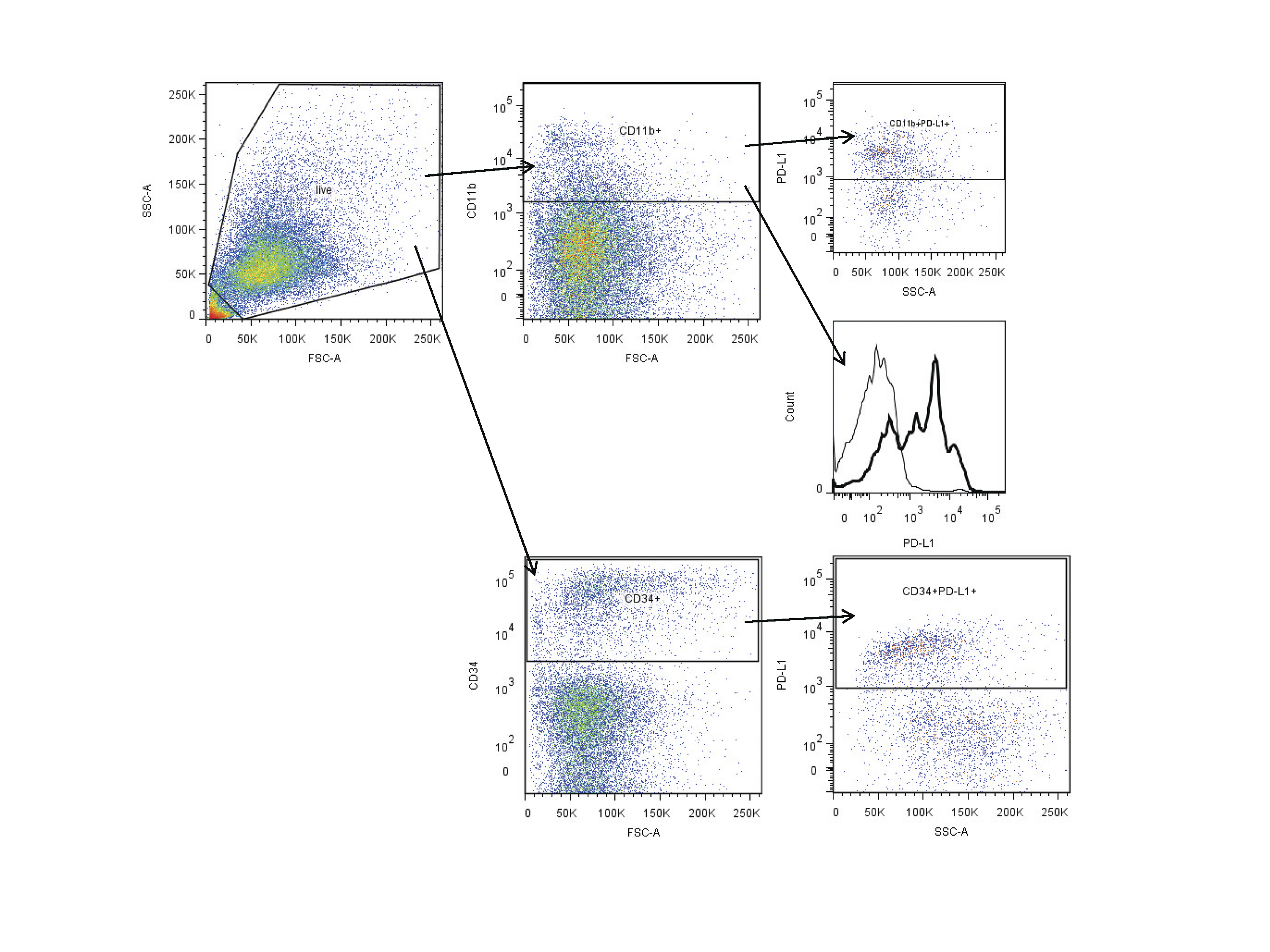

Supplement: Figure S3 — Strategy for gating CD11b and CD34 cells expressing PD-L1. The percentage of PD-L1 positive cells in the CD11b population was calculated as the percentage of PD-L1 positive cells inside the CD11b gate. PD-L1 MFI histograms were created from the CD11b gate, isotype control is shown as a thin line. The percentage of CD34+PD-L1+ of live cells was calculated as the percentage CD34+PD-L1+ cells in the “live” gate. (TIF) [file pone.0055818.s003.tif]

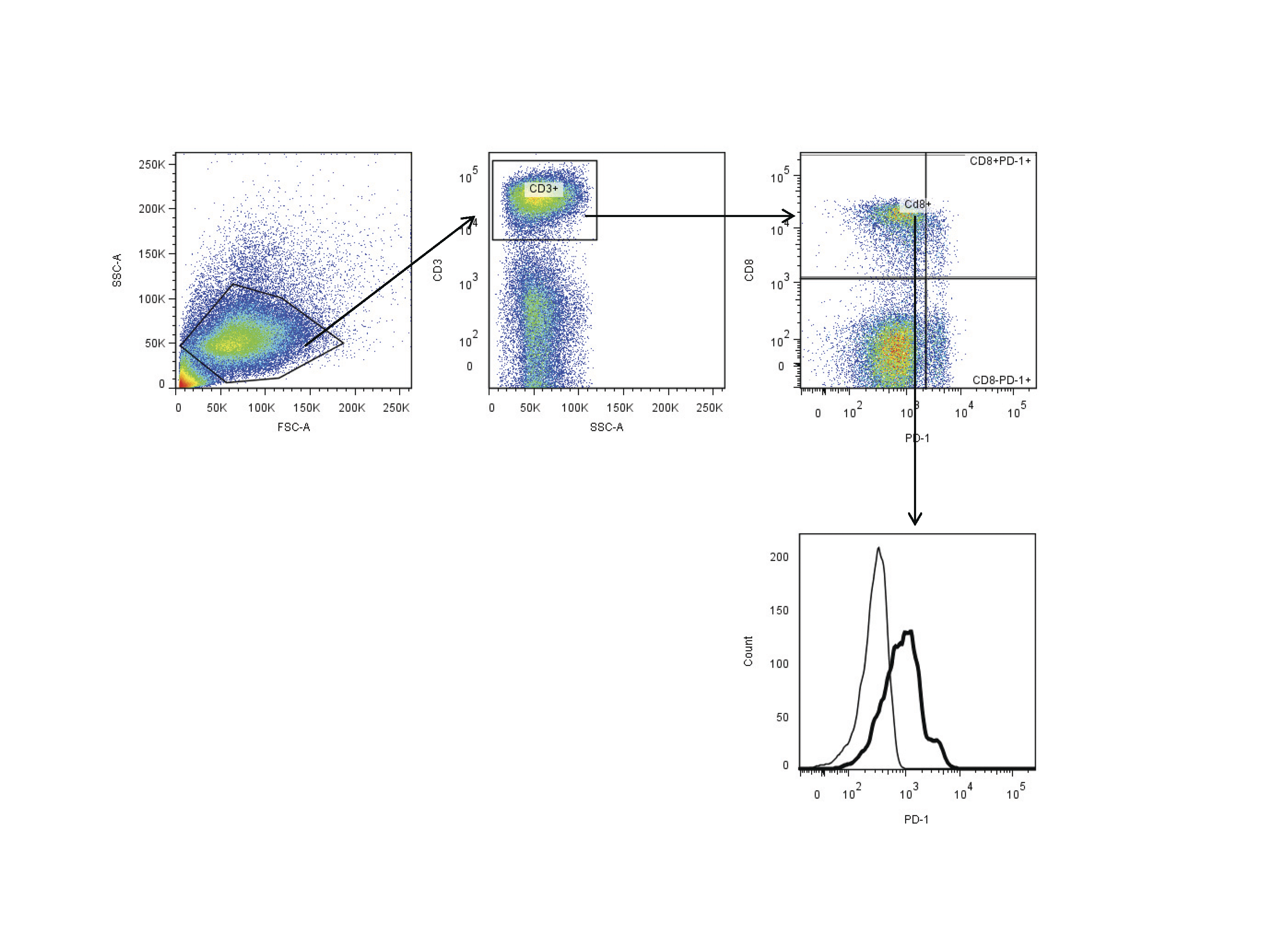

Supplement: Figure S4 — Strategy for gating PD-1 positive T cells. The percentage of PD-1 cells of CD8 cells was calculated as the percentage of PD-1 positive cells (right upper quadrant) of total CD8 cells (left and right upper quadrants). The percentage of PD-1 cells of CD8 negative cells was calculated as the percentage of PD1 positive cells (right lower quadrant) of total CD8 negative cells (left and right lower quadrant). PD-1 MFI histograms were created from CD8 cell gate (left and right upper quadrants), isotype control is shown as a thin line. (TIF) [file pone.0055818.s004.tif]

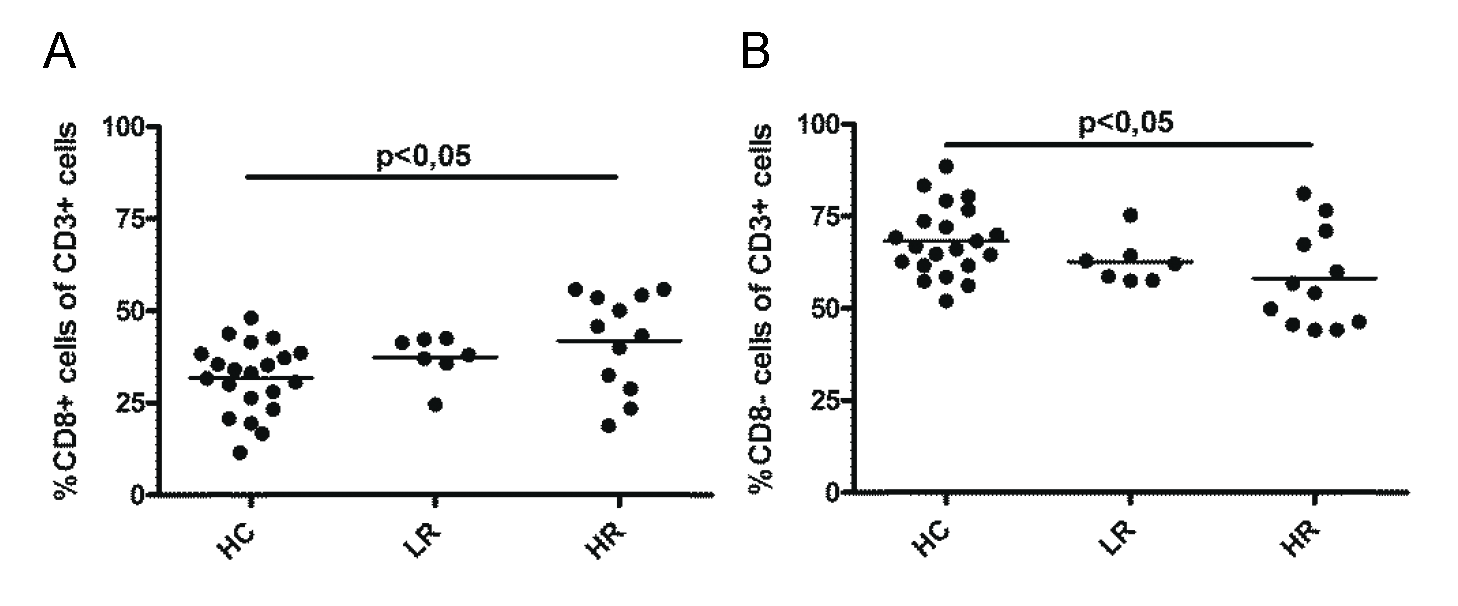

Supplement: Figure S5 — T cell levels. Level of CD8 (A) and CD8 negative (B) cells in LR (n = 7) and HR (n = 12) patients compared to HR (n = 21). Statistically significant differences between groups, reported as P-value in the figures were assessed by the non-parametric Kruskal Wallis test and Dunn's post test. (TIF) [file pone.0055818.s005.tif]

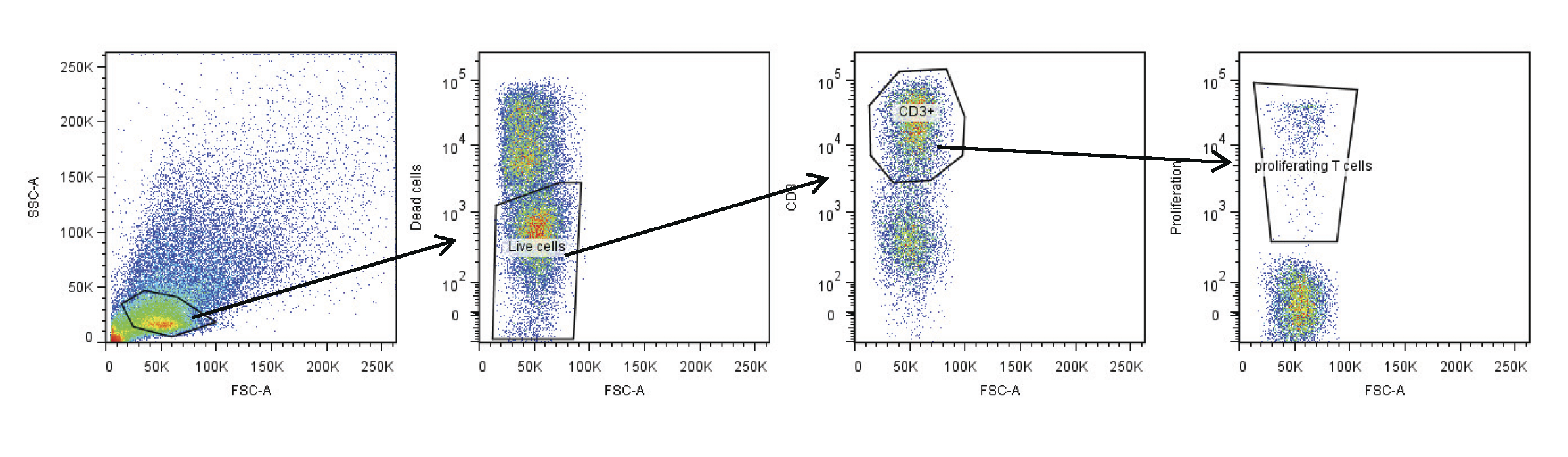

Supplement: Figure S6 — Strategy for gating proliferating T cells. The proliferating cells were calculated as the percentage of cells positive for the proliferation marker EdU in the CD3 gate. (TIF) [file pone.0055818.s006.tif]

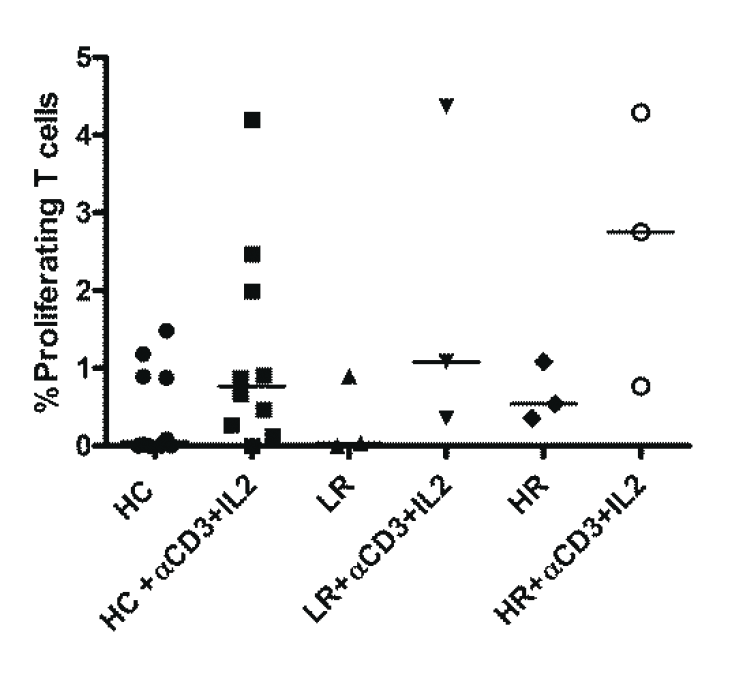

Supplement: Figure S7 — Proliferation of in vitro stimulated T cells. The percentage of proliferating T cells after in vitro stimulation of T cells from HCs (n = 10), LR (n = 3), HR (n = 3) patients. (TIF) [file pone.0055818.s007.tif]
